# Creation of standardized tools to evaluate reporting in health research: Population Reporting Of Gender, Race, Ethnicity & Sex (PROGRES)

**DOI:** 10.1371/journal.pgph.0002227

**Published:** 2023-09-07

**Authors:** Anne M. Stey, Mira Ghneim, Onaona Gurney, Ariel P. Santos, Rishi Rattan, Egide Abahuje, Archit Baskaran, Jeffry Nahmias, Joseph Richardson, Tanya L. Zakrison, Zinzi D. Baily, Elliott R. Haut, Mihir Chaudhary, Bellal Joseph, Ben Zarzaur, Kimberly Hendershot

**Affiliations:** 1 Department of Surgery, Feinberg School of Medicine, Northwestern University, Chicago, IL, United States of America; 2 R Adams Cowley Shock Trauma Center, University of Maryland School of Medicine, Baltimore, MD, United States of America; 3 Department of Surgery, New York University Langone Health, Brooklyn, NY, United States of America; 4 Department of Surgery, Texas Tech University Health Sciences Center, Lubbock, TX, United States of America; 5 DeWitt Daughtry Family Department of Surgery, University of Miami, Miami, FL, United States of America; 6 Department of Surgery, University of California, Irvine, Orange, CA, United States of America; 7 Department of African American Studies, University of Maryland, College Park, MD, United States of America; 8 Section of Trauma and Acute Care Surgery, University of Chicago, Chicago, IL, United States of America; 9 Soffer Clinical Research Center, University of Miami, Miami, FL, United States of America; 10 Department of Surgery, Division of Acute Care Surgery, Johns Hopkins University, Baltimore, MD, United States of America; 11 Department of Surgery, Division of Trauma, Critical Care, Emergency Surgery and Burns, College of Medicine, University of Arizona, Tucson, AZ, United States of America; 12 Department of Surgery, University of Wisconsin, Madison, WI, United States of America; 13 Department of Surgery, Division of Acute Care Surgery, University of Alabama Birmingham, Birmingham, AL, United States of America; University of Toronto Temerty Faculty of Medicine, CANADA

## Abstract

Despite increasing diversity in research recruitment, research finding reporting by gender, race, ethnicity, and sex has remained up to the discretion of authors. This study developped and piloted tools to standardize the inclusive reporting of gender, race, ethnicity, and sex in health research. A modified Delphi approach was used to develop standardized tools for the inclusive reporting of gender, race, ethnicity, and sex in health research. Health research, social epidemiology, sociology, and medical anthropology experts from 11 different universities participated in the Delphi process. The tools were pilot tested on 85 health research manuscripts in top health research journals to determine inter-rater reliability of the tools. The tools each spanned five dimensions for both sex and gender as well as race and ethnicity: Author inclusiveness, Participant inclusiveness, Nomenclature reporting, Descriptive reporting, and Outcomes reporting for each subpopulation. The sex and gender tool had a median score of 6 and a range of 1–15 out of 16 possible points. The percent agreement between reviewers piloting the sex and gender tool was 82%. The interrater reliability or average Cohen’s Kappa was 0^.^54 with a standard deviation of 0^.^33 demonstrating moderate agreement. The race and ethnicity tool had a median score of 1 and a range of 0–15 out of 16 possible points. Race and ethnicity were both reported in only 25^.^8% of studies evaluated. Most studies that reported race reported only the largest subgroups; White, Black, and Latinx. The percent agreement between reviewers piloting the race and ethnicity tool was 84 and average Cohen’s Kappa was 0^.^61 with a standard deviation of 0^.^38 demonstrating substantial agreement. While the overall dimension scores were low (indicating low inclusivity), the interrater reliability measures indicated moderate to substantial agreement for the respective tools. Efforts in recruitment alone will not provide more inclusive literature without improving reporting.

## Introduction

Over the last decade, there has been a concerted effort to address noticeable gaps in participant representation in health research. For instance, the National Institutes of Health (NIH)–the nation’s primary medical research agency–have launched initiatives, such as “All of US,” which seek to improve the diversity of research participants [1]. Additionally, all NIH funded research must attempt to recruit diverse participants to produce results generalizable to the broader population [2]. The ultimate goal of scientific health research at large is to produce greater diversity, transparency, accessibility, and generalizability of health research data [3]. However, despite increasing diversity in recruitment and research participation, the ultimate reporting of research findings by gender, race, ethnicity, and sex has remained unspecified and up to the discretion of authors [4].

The Enhancing the QUAlity and Transparency Of health Research (EQUATOR) Network is a global initiative that has devised standardized reporting guidelines for most types of biomedical research study designs (i.e. randomized trials, systematic reviews, and observational studies) [5]. The EQUATOR mission is to achieve accurate, complete and transparent reporting of all health research studies to support research reproducibility and usefulness [1]. These guidelines have been adopted by scientific journals, such as the JAMA network, and serve as a reference for scientific health research reporting [6,7]. However, no EQUATOR guidelines exist for the standardized reporting of gender, race, ethnicity, and sex.

Many published calls to action shed light on the poor reporting of gender, race, ethnicity, and sex, including one by the Eastern Association for the Surgery of Trauma (EAST) with multidisciplinary colleagues representing clinician researchers, social epidemiologists, sociologists, and medical anthropologists [8]. Poor reporting prevents better understanding of health outcomes in marginalized populations, such as immigrant, Black, and Indigenous people [9]. No study to date has suggested how to operationalize standardized reporting. This study sought to develop and then pilot tools to standardize the inclusive reporting of gender, race, ethnicity, and sex in health research.

## Methods

This study used a modified Delphi approach to develop a standardized tool for the inclusive reporting of gender, race, ethnicity, and sex in health research. The subsequent tools were pilot tested on health research manuscripts in four top health research journals to determine inter-rater reliability of the tools.

### Expert convening

Health research, social epidemiology, sociology, and medical anthropology experts were identified from the EAST professional society and beyond. Initially 30 Individuals from the Multicenter Trials and the Equity, Quality, and Inclusion in Trauma Surgery Practice Committees were invited to join. A total of 15 experts from 11 different universities representing all regions of the United States agreed to participate. The participants and non-participants were similar in age, gender, ethnicity, sex, academic and clinical background (S1 Table).

### Literature review

The aim of the literature review was to ascertain the breadth of categories for Gender, Race, Ethnicity and Sex in the English health research evidence. A literature search was performed querying Medline for the search terms “sex reporting” “gender reporting” “race reporting” and “ethnicity reporting”. There were no Mesh terms utilized. A time frame from 1960 to September 2020 was searched to chronicle the evolution of demographic categorizations over time. We limited the search terms to these four terms because of the long study period we were considering and the huge volume of articles that we chose to prioritize specificity as opposed to sensitivity of our search. A librarian assisted us in developing the search terms and pulling the articles. Only English-language peer-reviewed journal articles were included. The 15 individuals were divided into three groups of five people, one that screened and extracted data for sex and gender literature, one group that screened and extracted data for race literature, and one group that screened and extracted data for ethnicity literature. All members of each group independently screened titles, abstracts, and full text manuscripts. Discussion among all five members of each of the three groups was used to reach consensus for final full text inclusion for extraction. Reference lists from included full text publications were screened to identify other relevant literature.

### Extraction and statement development

Data regarding reporting equity in full text manuscripts were extracted into a Microsoft Word document. Extracted data were ranked according to level of evidence. The level of evidence in the available literature ranged from systematic reviews to expert opinion. The entire group reviewed the document and met twice as a group to achieve a draft statement consensus document summarizing the findings and recommendations based on the 19 full-texts included in the review [8].

### Tool development

The draft statement consensus document contained a series of recommendations for improved reporting equity based on the literature review [8]. Tools to assess the quality of reporting of gender, race, ethnicity, and sex in published manuscripts was devised by two individuals (AS,KH) based on a three-step modified Delphi consensus process which took place between September to December 2020 [10]. A comprehensive list of five dimensions was drafted after expert participants reviewed the existing literature. This list was iteratively reviewed using systematic progression of repeated rounds of voting to determine expert group consensus. The modified Delphi consensus process consisted of two rounds of emails and one virtual meeting. The tools quantified reporting along five dimensions: authorship inclusiveness, participant inclusiveness, nomenclature, descriptive reporting, and outcome reporting. A comprehensive list of potential components organized into these five dimensions was drafted. Each dimension specified 1–2 components. This list was iteratively reviewed using systematic progression of repeated rounds of voting to determine expert group consensus. The modified Delphi consensus process consisted of one round of emails and one virtual meeting.

#### Round 1

The tool was circulated via email to the 15 participants with the goal to optimize the tools’ syntax. Each individual was asked to review and agree or disagree with each component in the sex and gender tool as well as the race and ethnicity tool. Responses were gathered via email. Components required 80% agreement to be accepted or omitted. Components that did not reach 80% threshold for acceptance on the first round were adapted via participant input and redistributed in the second round.

#### Round 2

The same consensus method was used in this round but accomplished via a virtual meeting. Participants were encouraged to discuss the entire tools and all components until agreement was reached to retain, modify, or eliminate components. Responses were collated and analyzed and the tool, adapted based on recommendations from participants.

#### Round 3

The tool in its entirety was then circulated again via email. The same consensus method was used as in round 1. Final responses were analyzed and described with components reaching 80% agreement being retained in the final tools.

### Pilot testing

Using the tools, seven individual from the 15 experts assessed the 66 published original research reports from December 2020 in four of the highest impact health research journals: the New England Journal of Medicine, Journal of the American Medical Association, the British Medical Journal, and the Lancet (S2 Table). Additionally, the tools were piloted utilizing the manuscripts from 19 Multicenter Trials supported by the Eastern Association for the Surgery of Trauma, the Western Trauma Association, and the American Association for the Surgery of Trauma from January 2019-January 2021 to oversample reports from a field of health research where participants and patients are disproportionately from marginalized racial and ethnic groups. There was no deliberate oversampling of original research focused on specific racial and ethnic populations (e.g., the Sister Study, Black Women’s Health Study). Each published report was reviewed for gender, race, ethnicity, and sex reporting in each of five dimensions. Author inclusiveness reporting was assessed based on personal knowledge of authors, or internet web search for gender, race, ethnicity, and sex identification. Participant inclusiveness reporting was assessed based on whether multiple gender, race, ethnicity, and sex were recruited or included. Nomenclature reporting was assessed based on the utilization of specific gender, race, ethnicity, and sex categories specified by the US census [11]. Descriptive reporting was assessed based on whether the results section or descriptive tables presented data by gender, race, ethnicity, and sex composition. Outcomes reporting was assessed based on whether the results section or univariate and multivariate outcomes tables reported data for each subpopulation separately.

Two reviewers scored each study independently using the tools in blinded fashion. Each study was scored based on inclusion or exclusion of each component within the sex and gender tool (S1 Data) and the race and ethnicity tool, respectively (S2 Data). Interrater reliability (measured as percent agreement and Cohen’s kappa) was calculated between the two reviewers for each component of each tool. Components with less than 90% agreement were excluded from the final tool. Interrater reliability was also assessed for each of the overall tools.

## Results

Two tools emerged from the review of literature and Delphi process. The tools each spanned five dimensions for both sex and gender (Table 1) as well as race and ethnicity (Table 2). Author inclusiveness reporting assessed whether studies had diverse authorship, which may influence analysis, interpretation, and results reporting. Participant inclusiveness reporting assessed whether a broad range of participants were recruited. Nomenclature reporting assessed how granular gender, race, ethnicity, and sex were captured. By granular, we mean how detailed were gender, race, ethnicity and sex reported. Descriptive reporting assessed whether descriptive statistics included gender, race, ethnicity, and sex composition. Outcomes reporting assessed whether univariate and multivariate outcomes were reported separately for each subpopulation. The tools did not consider the assessment of interactions, stratified or sensitivity analyses.

**Table 1:** Sex and gender reporting tool generated from the delphi process.

	Dimension	Description		Points
1	**Authorship inclusiveness**	Author representation of different sexes and genders		1
2	**Participant inclusiveness**	Participant inclusion of different sexes		1
		Participant inclusion of different genders		1
		Why one or both not applicable		
3	**Nomenclature**	**Gender**	Men	1
			Women	1
			Transgender	1
			Non-binary/Non-conforming	1
		**Sex**	Male	1
			Female	1
			Inter-sex	1
4	**Descriptive reporting**	By sex		1
		By gender		1
5	**Outcome reporting**	Univariate outcomes by sex		1
		Multivariate outcomes by sex		1
		Univariate outcomes by gender		1
		Multivariate outcomes by gender		1
	**Total Points**			16

**Table 2:** Race and ethnicity reporting tool generated from the delphi process.

	Dimensions	Description		Points
**1**	**Author inclusiveness**	Author representation of different races and ethnicities		1
**2**	**Participant inclusiveness**	Participant inclusion of different races		1
		Participant inclusion of different ethnicities Or why one or both not applicable		1
**3**	**Nomenclature**	**Race**	Native American or Alaska Native	1
			Asian	1
			Black	1
			Native Hawaiian or Pacific Islander	1
			White	1
		**Ethnicity**	Hispanic/Latinx *	1
			Classification beyond Hispanic**	1
**4**	**Descriptive reporting**	By race		1
		By ethnicity		1
**5**	**Outcome reporting**	Univariate outcomes by race		1
		Multivariate outcomes by race		1
		Univariate outcomes by ethnicity		1
		Multivariate outcomes by ethnicity		1
	**Total Points**			**16**

* NIH Standard Definition

** Supplemental table.

Sex and gender reporting components were each dichotomous and ranged from 0 to 1 in the 85 published manuscripts in peer-reviewed journals. The sex and gender tool had a median score of 6 and a range of 1–15 out of 16 possible points. All studies captured sex or gender in at least one dimension of the tool. However, there was repeated conflation between biologic sex and self-reported gender, and sexual orientation (not explicitly assessed in this tool), and no studies reporting both sex and gender. There was extremely low reporting of non-binary people, transgender men, transgender women, and intersex people. If transgender people were included, most studies collapsed it into one single monolithic label without differentiation between patients’ specific identities within the transgender community (ex. transmasculine, transfeminine, transgender woman, transgender man, etc). Despite these challenges, the overall percent agreement between reviewers piloting the sex and gender tool was 82%. The interrater reliability or average Cohen’s Kappa for all reviewers reviewing all studies with the sex and gender tool was 0^.^54 with a standard deviation of 0^.^33 demonstrating moderate agreement.

The race and ethnicity tool had a median score of 1 and a range of 0–15 out of 16 possible points. Race and ethnicity were both reported in only 25^.^8% of the high impact studies evaluated. Most studies that reported race reported only the largest subgroups such as White, Black, and Latinx. Despite these challenges, the overall percent agreement between reviewers piloting the tool for the race and ethnicity tool was 84%. The interrater reliability or average Cohen’s Kappa for the reviewers reviewing all studies with the race and ethnicity tool was 0^.^61 with a standard deviation of 0^.^38 demonstrating substantial agreement.

## Discussion

In our study, we were able to develop and pilot a tool to standardize the inclusive reporting of gender, race, ethnicity, and sex in health research. Despite recent initiatives in trying to capture inclusive study populations, there appears to be a lack of granular reporting of data for specific marginalized populations who are typically burdened disproportionately by negative health outcomes [4]. Poor reporting prevents better understanding of health outcomes in marginalized populations, such as transgender, Black, and Indigenous people [9]. There have been previous calls for more standardized reporting of gender, race, ethnicity and sex [12,13]. This study sought to develop a tool for standardized reporting of gender, race, ethnicity, and sex in health research. As such, we developed and piloted a joint reporting tool which demonstrated that recent literature had better reporting of dimensions of sex and gender than for dimensions of race and ethnicity. The interrater reliability was moderate for the sex and gender reporting and substantial for the race and ethnicity reporting tool.

The results of this study compares well with prior studies that demonstrate a great degree of heterogeneity in the reporting of gender, race, ethnicity [14], and sex [15,16]. There have been calls for improved reporting across all of health research ranging from alcohol-use disorders to cancer over the last two decades [17,18]. However, to our knowledge this is the first study that has developed and piloted of a tool to standardize gender, race, ethnicity, and sex reporting in health research. If further validated, this reporting tool could be particularly powerful upon adoption by the EQUATOR network by allowing more widespread adoption for standardized reporting of gender, race, ethnicity, and sex in all disciplines of health research.

There are several limitations to this study. First, the expert panel convened included few researchers from other disciplines and could have included more people from under-represented and marginalized groups. Although we attempted to adhere to established gender, race, ethnicity, and sex categories based on the United States Census, and the National Institutes of Health [19], we seek input to revise and make more inclusive tools to better capture our diverse population (Table 3). Second, these tools have not been extensively validated by raters who were not part of this panel. This will be an important future direction. Third, pilot testing with manuscripts from the four highest impact health research journals puts the findings at risk for selection bias due to bias in what is accepted for publication in high impact health journals. However, we attempted to overcome this limitation by also including manuscripts from several multicenter trials published in a variety of journals. Fourth, author names, personal knowledge of authors, and web searches were used to give points for authorship inclusiveness scoring. However, this approach would not be easily replicated in the broader literature because names and web pages may not reflect authors’ gender, race, ethnic, or sex identity. We recommend that upon implementation of these tools, authors should be asked to self-identify this information. Furthermore, we encourage journal to routinely collected these data upon submission and publication to assess journal-specific author inclusivity over time. Fifth, race and ethnicity are social constructs, and our conceptualization is based on a United States-centric approach to race and ethnicity. For instance, the race and ethnicity reporting tool was focused on overall populations with known disparities in care within the United States, while recognizing that the large categories hide wide heterogeneity (e.g., different ethnicities within the Asian or Black category). We recommend that the race and ethnicity tool be contextually adapted for regions when applied internationally in order to accurately capture local ethnicities facing disparities in health and a disproportionate burden of social determinants of health inequities.

**Table 3:** Ethnic Ancestry Categories in the United States [19].

**Afghan** **African** **African American** **Albanian** **American** **American Indian** **Arab** **Argentinean** **Armenian** **Asian** **Asian Indian** **Austrian**	**Bangladeshi** **Barbadian** **Belgian** **Bhutanese** **Brazilian** **British** **Cambodian** **Canadian** **Chilean** **Chinese** **Colombian** **Costa Rican**	**Croatian** **Cuban** **Cypriot (Greek and Turkish)** **Czech** **Czechoslovakian** **Danish** **Dominican** **Dutch** **Ecuadorian** **Ethiopian** **English**	**Filipino** **Finnish** **French** **French Canadian** **Georgian** **German** **Greek** **Guatemalan** **Guyanese** **Haitian** **Hispanic** **Hmong**	**Honduran** **Hungarian** **Icelandic** **Indian** **Indonesian** **Iranian** **Irish** **Israeli** **Italian** **Jamaican** **Japanese** **Korean**
**Laotian** **Latin American** **Lithuanian** **Malaysian** **Mexican** **Nicaraguan** **Nigerian** **Nepali** **Norwegian** **Pakistani** **Panamanian**	**Peruvian** **Polish** **Portuguese** **Puerto Rican** **Romanian** **Russian** **Salvadorian** **Samoan** **Scandinavian** **Scottish Irish** **Scottish**	**Serbian** **Sikh** **Sri Lankan** **Slavic** **Slovak** **Slovene** **Spaniard** **Swedish** **Swiss** **Taiwanese** **Thai**	**Trinidadian and Tobagonian** **Turkish** **Ukrainian** **Venezuelan** **Vietnamese** **Welsh** **West Indian** **Yugoslavian**	

There remains a great deal of opportunity to ensure a more inclusive recruitment and enrollment of diverse and marginalized populations within health research. We believe this will translate into improved methodological analyses and reporting of important research findings. This study involved the design and piloting of a tool for standardized reporting of gender, race, ethnicity, and sex in health research. While the overall dimension scores were relatively low (indicating low inclusivity), the interrater reliability measures indicated moderate to substantial agreement for the respective tools. All the efforts in recruitment and enrollment will not alone provide richer, more inclusive scientific health research literature without improving detailed reporting of data involving under-represented and marginalized populations.

## Supporting information

S1 Table Demographic characteristics of experts who participated in the delphi process. Legend: This table denotes the aggregate age, gender, and ethnicitiy of participants of the literature review and delphi process.
(DOCX)

S2 Table Manuscripts reviewed for piloting gender race ethnicity and sex reporting tool. Legend: This table contains the title, journal and link to articles in top health journals used to pilot the gender race ethnicity and sex reporting tools.
(XLSX)

S1 Data Gender and sex reporting tool data collected. Articles with gender and sex reporting rating by study team with analytic macro of concordance.
(XLSM)

S2 Data Race and ethnicity reporting tool data collected. Legend: Articles with race and ethnicity reporting rating by study team with analytic macro of concordance.
(XLSM)
