# Supplementary material for: Creation of standardized tools to evaluate reporting in health research: Population Reporting Of Gender, Race, Ethnicity & Sex (PROGRES)
Source: PLOS Glob Public Health. 2023 Sep 7;3(9):e0002227. doi: 10.1371/journal.pgph.0002227 (PMC10484436; doi:10.1371/journal.pgph.0002227)
Supplement: S1 Table — Legend: This table denotes the aggregate age, gender, and ethnicitiy of participants of the literature review and delphi process. (DOCX) [file pgph.0002227.s001.docx]

|  | **Participants N =15** | **Non-participants N =15** | **P-Value** |
| --- | --- | --- | --- |
|  | n (%) | n (%) |  |
| **Gender** |  |  |  |
| Woman | 6 (40) | 7 (46.7) | 0.73 |
| Man | 9 (60) | 8 (53.3) |  |
| Non-binary/Non-conforming | 0 | 0 |  |
| **Race** |  |  |  |
| Native American/Alaska Native |  |  |  |
| Asian | 4 (26.7) | 3 (20.0) | 0.44 |
| Black | 2 (13.3) | 2 (13.3) |  |
| Native Hawaiian/ Pacific Islander | 2 (13.3) | 0 |  |
| White | 7 (46.7) | 10 (66.7) |  |
| **Ethnicity** |  |  |  |
| Hispanic/Latinx * | 1 (6.7) | 3 (80) | 0.28 |
| Classification beyond Hispanic** | 14 (93.3) | 12 (20) |  |
| **Sex** |  |  |  |
| Female | 6 (40) | 7 (46.7) | 0.71 |
| Male | 9 (60) | 8 (54.3) |  |
| Inter-sex |  |  |  |
| **Age (years)** |  |  |  |
| <40 | 4 (26.7) | 1 (6.7) | 0.33 |
| 40-60 | 10 (66.7) | 13 (86.6) |  |
| >60 | 1 (6.6) | 1 (6.7) |  |
| **Academic Background** |  |  |  |
| MD | 13 (86.7) | 15 (100) | 0.14 |
| PhD | 2 (13.3) | 0 |  |
| **Clinical Background** |  |  |  |
| Surgery | 14 (93.3) | 14 (93.3) | 1.00 |
| Medicine | 1 (6.7) | 1 (6.7) |  |

**Appendix: Demographic Characteristics of Experts who participated in the Delphi Process**

This table displays demographic characteristics of experts who were invited to participate in the Delphi Process. Participants are those who responded and agreed to participate, non-participants are those who were invited but did not participate in the Delphi Process. Demographic characteristics of participants were not statically significant different from those of who did not participate.
